# How the Pathogenic Fungus *Alternaria alternata* Copes with Stress via the Response Regulators SSK1 and SHO1

**DOI:** 10.1371/journal.pone.0149153

**Published:** 2016-02-10

**Authors:** Pei-Ling Yu, Li-Hung Chen, Kuang-Ren Chung

**Affiliations:** Department of Plant Pathology, College of Agriculture and Natural Resources, National Chung-Hsing University, Taichung, Taiwan; University of California, UNITED STATES

## Abstract

The tangerine pathotype of *Alternaria alternata* is a necrotrophic fungal pathogen causing brown spot disease on a number of citrus cultivars. To better understand the dynamics of signal regulation leading to oxidative and osmotic stress response and fungal infection on citrus, phenotypic characterization of the yeast SSK1 response regulator homolog was performed. It was determined that SSK1 responds to diverse environmental stimuli and plays a critical role in fungal pathogenesis. Experiments to determine the phenotypes resulting from the loss of SSK1 reveal that the *SSK1* gene product may be fulfilling similar regulatory roles in signaling pathways involving a HOG1 MAP kinase during ROS resistance, osmotic resistance, fungicide sensitivity and fungal virulence. The *SSK1* mutants display elevated sensitivity to oxidants, fail to detoxify H_2_O_2_ effectively, induce minor necrosis on susceptible citrus leaves, and displays resistance to dicarboximide and phenylpyrrole fungicides. Unlike the SKN7 response regulator, SSK1 and HOG1 confer resistance to salt-induced osmotic stress via an unknown kinase sensor rather than the “two component” histidine kinase HSK1. SSK1 and HOG1 play a moderate role in sugar-induced osmotic stress. We also show that SSK1 mutants are impaired in their ability to produce germ tubes from conidia, indicating a role for the gene product in cell differentiation. SSK1 also is involved in multi-drug resistance. However, deletion of the yeast *SHO1* (*s*ynthetic *h*igh *o*smolarity) homolog resulted in no noticeable phenotypes. Nonetheless, our results show that *A*. *alternata* can sense and react to different types of stress via SSK1, HOG1 and SKN7 in a cooperative manner leading to proper physiological and pathological functions.

## Introduction

Plant pathogenic fungi deploy different strategies to attack their hosts. One of the weapons used by fungal pathogens to colonize host plants is to produce toxins that kill host cells. Toxic reactive oxygen species (ROS) are released and osmotic imbalance could occur as a result of plant cell death. Thus, a successful pathogen must be able to deal with oxidative and osmotic stress in order to survive and successfully colonize within the acrimonious environment. The tangerine pathotype of *Alternaria alternata* produces the host-selective ACT toxin, which has been demonstrated to be essential for fungal pathogenesis [1,2]. The ability to tolerate oxidative stress also is vital for *A*. *alternata* pathogenesis in citrus [3,4].

Several cellular regulators and key enzymes, including the YAP1 transcription regulator, the NADPH oxidase (NOX), the HOG1 MAP kinase, the SKN7 response regulator, and the nonribosomal peptide synthetase (NPS6) and the glutathione peroxidase (GPx3) have been shown to be required for ROS tolerance and full virulence in citrus in *A*. *alternata* [5–12]. The ability to cope with osmotic stress via the HOG1 MAP kinase and the SKN7 response regulator has also been shown to be required for *A*. *alternata* pathogenesis in citrus [7,8]. In the budding yeast *Saccharomyces cerevisiae*, both HOG1 and SKN7 are downstream regulators of “two-component” histidine kinase HSK1 involved in osmotic adaptation [13,14]. In the tangerine pathotype of *A*. *alternata*, HSK1 and SKN7 are the primary regulators of resistance to sugar but not salt-induced osmotic stress [7]. In contrast, HOG1, perhaps acting in parallel with SKN7, is primarily responsible for salt-induced osmotic stress.

In *A*. *alternata*, both HOG1 and SKN7 also are involved in oxidative stress resistance and fungal pathogenesis while HSK1 plays no roles at all in these functions. The roles of SKN7 and HOG1 in the oxidative stress response are apparently independent of the HSK1-mediatd regulation. Moreover, SKN7 is required for resistance to peroxides but not superoxide because mutant strains defective for SKN7 are hypersensitive to H_2_O_2_, cumyl peroxide and *tert*-butyl hydroperoxide, but not to the superoxide-generating compounds―potassium superoxide, menadione and diamide [8]. In contrast, HOG1 is required for resistance to a wide array of oxidants. Apart from oxidative and osmotic stress, fungi carrying HOG1 or SKN7 deletions are slightly more resistant to dicarboximide (iprodione and vinclozolin) or phenylpyrrole (fludioxonil) fungicides compared to wild-type. Fungal mutants defective for HSK1 or both SKN7 and HOG1 (*skn7 hog1* double mutant) are highly resistant to these fungicides. It appears that *A*. *alternata* can sense and react to different types of stress via HSK1, SKN7 and/or HOG1 [3].

Microorganisms such as *S*. *cerevisiae* utilize a histidine kinase (HSK1)-mediated phosphorelay system between histidine (His) and aspartate (Asp) to sense and adapt to environmental changes [15–17]. In *S*. *cerevisiae*, HSK1 is autophosphorylated under low osmotic conditions. The phosphate is transduced down to a histidine-containing protein (HPt) and subsequently to two response regulators—SSK1 (an upstream regulator of HOG1) and SKN7. The phosphorylated SKN7 can regulate genes associated with low osmolarity. In contrast, phosphorylated SSK1 is inactive under low osmotic conditions. Under high osmotic conditions, unphosphorylated SSK1 activates HOG1-mediated gene expression. SSK1 response regulator is the central component of the histidine kinase-MAPK phosphorelay system in *S*. *cerevisiae* [18]. In filamentous fungi, SSK1 also is an important regulator for integration of signaling transduction and plays a pivotal role in stress response and/or fungal penetration and colonization [19–21]. In addition to HSK1, activation of the HOG1 pathway also is regulated by a SHO1 (*s*ynthetic *h*igh *o*smolarity) signaling protein in *S*. *cerevisiae* [22–24].

Judging from phenotypic commonalities between *HSK1* and *SKN7* mutants, *A*. *alternata* copes with sugar-induced osmotic stress likely via HSK1 and SKN7. HOG1, independent of HSK1 and SKN7, is involved in salt-induced osmotic stress. The requirement of SKN7 in the oxidative stress response appears to be independent of HSK1 and HOG1, implicating the existence of different regulatory pathways in response to osmotic and oxidative stress. Both SKN7 and HOG1 are required for infection of citrus while HSK1 plays no role in pathogenesis. To delineate the complexity of HSK1, SKN7 and HOG1 involving in coordinating fungal response to different types of environmental stress and pathogenesis, we sought to determine the functions of the *SSK1* homolog by creating and analyzing loss-of-function mutants. We also functionally characterized the *SHO1* homolog, revealing that, unlike SSK1, SHO1 plays no roles in oxidative and osmotic stress and fungal virulence in the tangerine pathotype of *A*. *alternata*.

## Results

### Characterization of *A*. *alternata SSK1* and *SHO1*

The full-length *SSK1* (*AaSSK1*) and *SHO1* (*AaSHO1*) gene sequences were amplified by PCR from genomic DNA of the tangerine pathotype of *A*. *alternata*. The *AaSSK1* gene contains a 2408-bp open reading frame (ORF) interrupted by a 50-bp intron, which encodes a polypeptide of 785 amino acids. AaSSK1 displayed strong similarity to the SSK1 response regulators of fungi. A stress responsive element (STRE: AGAGGGG) that is commonly found in genes induced by oxidative damage in yeasts was found in the 5’ untranslated region of *AaSSK1*. Analysis of the AaSSK1 polypeptide identified a signal receiver domain (a.a. 536 to 590), which is likely involved in receiving a signal from a sensor partner in a “two-component” histidine kinase-mediated system. The asparagine present at 539 is the putative site for phosphorylation. Two recognition sites are present between amino acids 586 to 587 (LE) and 589 to 591 (TKE). Amino acids 676 to 678 (WLE) were likely involved in intermolecular dimerization.

The *A*. *alternata SHO1* (*AaSHO1*) gene has a 1165-bp ORF interrupted with three introns of 65, 57, and 59 bp, which encodes a polypeptide of 327 amino acids. Unlike AaSSK1, no stress responsive element (STRE: AGAGGGG) was found in the promoter region of *AaSHO1*. AaSHO1 contains a Src homology 3 (SH3) domain (a.a. 254 to 308) found in the high osmolarity signaling protein Sho1p of *S*. *cerevisiae*.

### Disruption of *AaSSK1* Impacts Vegetative Growth, Conidial Germination, Differentiation and Protoplast Formation

**Fig 1:**
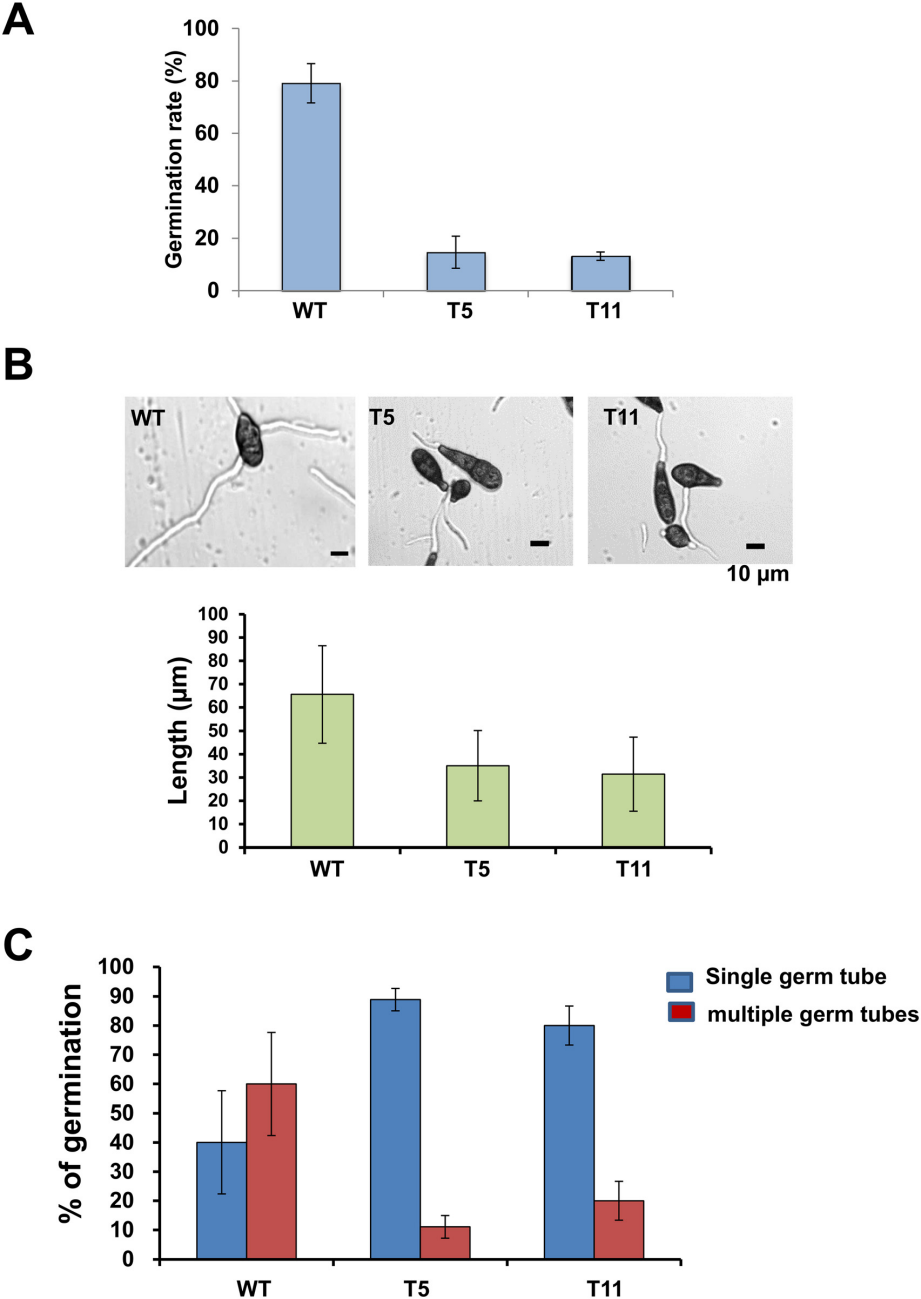
The *A*. *alternata SSK1* homolog (*AaSSK1*) is required for conidial germination and differentiation. **(A)** Percentage of conidial germination of the wild-type (WT) and the Δssk1 deletion mutants (T5 and T11) on 96-well microtiter plates for 6 h. **(B)** Images of germinated conidia (top panel) and quantitative analysis of the length of germ tubes on glass slides (bottom panel). **(C)** Production of single or multiple germ tubes on glass slides. All (100%) conidia prepared from WT and Δssk1 germinated on glass slides at 6 h. The data presented are the mean and standard deviation of two independent experiments with at least three replicates.

A split marker gene deletion allowed the identification of two *A*. *alternata* mutants (T5 and T11) defective at *SSK1* locus (S1 Fig). The *AaSSK1* impaired mutants reduced radial growth by 8% compared to wild-type cultured on PDA. Δssk1 mutants produced abundant ovoid conidia with both cross and longitudinal septae with dark pigment closely resemble those produced by wild-type on PDA. Conidia prepared from Δssk1 mutants germinated at rates and magnitudes much slower than those of wild-type when assayed on 96-well microtiter plates (Fig 1A). More than 80% of wild-type conidia germinated while less than 20% of conidia prepared from Δssk1 mutants germinated on microtiter plates. Moreover, the length of germ tubes produced from mutant conidia was 50% shorter than those of wild-type (Fig 1B). Conidia collected from wild-type produced multiple germ tubes; less than 50% of wild-type conidia examined produced single germ tubes. In contrast, the majority (>80%) of Δssk1 mutant conidia produced single germ tubes (Fig 1C), indicating that mutation of *SSK1* affected the pattern of conidial germination. Moreover, wild-type strain treated with cell wall degrading enzymes (CWDEs) resulted in protoplasts at quantities greater than 5 x10^6^ protoplasts per ml. Neither Δssk1-T5 nor Δssk1-T11 treated with CWDEs was able to generate any viable protoplasts, forming numerous broken cell fragments (data not shown) indicative of cell autolysis.

### AaSSK1 Is Required for Resistance to Oxidative Stress

**Fig 2:**
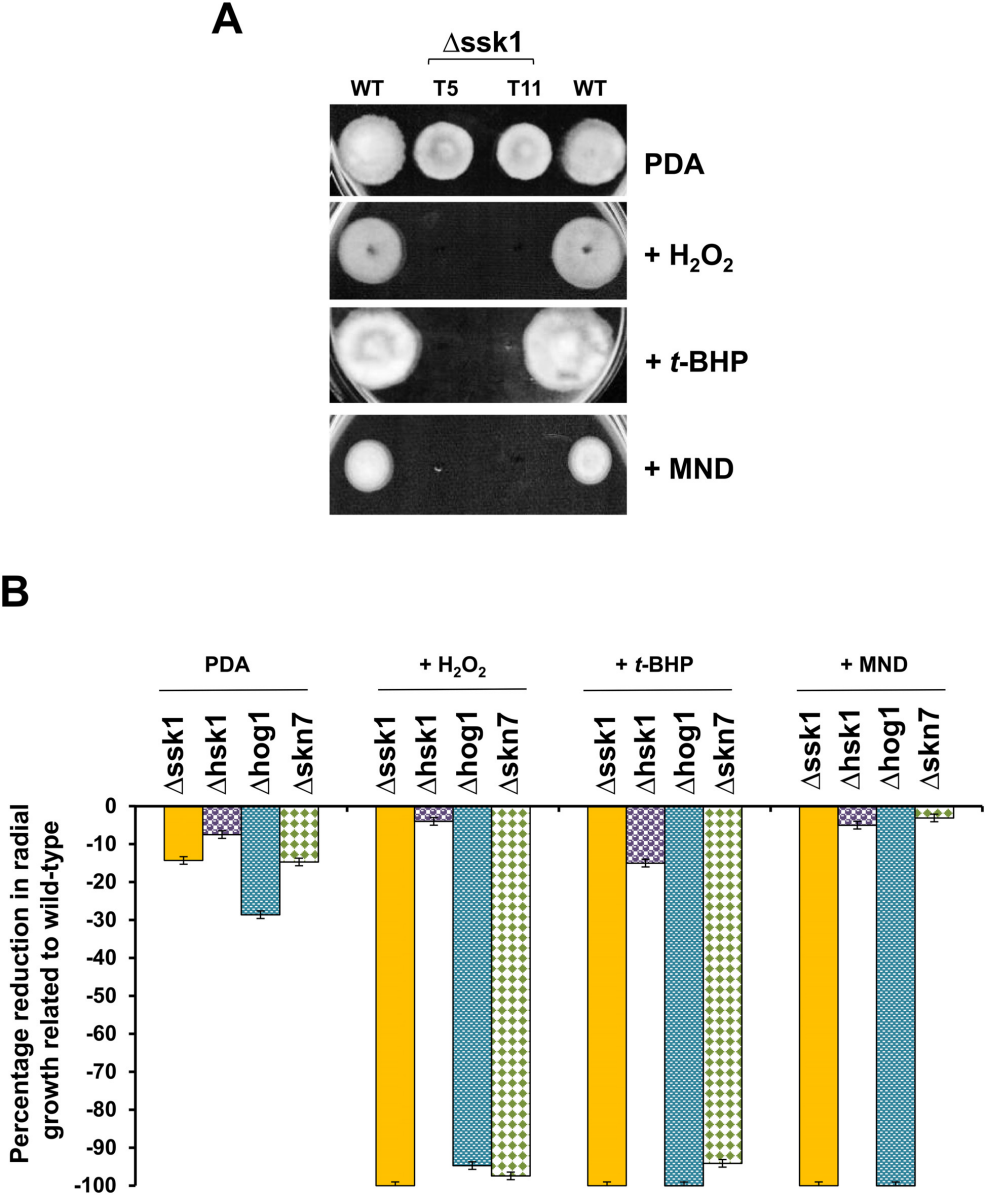
SSK1 is required for oxidative stress resistance in *A*. *alternata*. **(A)** Images of the wild-type (WT) and the Δssk1 deletion mutants (T5 and T11) grown potato dextrose agar (PDA) amended with hydrogen peroxide (H_2_O_2_, 5 mM), *tert*-butyl-hydroxyperoxide (*t*-BHP, 2.6 mM) and menadione (MND, 1 mM) for 4 to 5 days. **(B)** Quantitative comparison of Δssk1 with different mutant strains—Δhsk1 impaired for histidine kinase, Δhog1 for HOG1 MAP kinase, and Δskn7 for SKN7 response regulator—in the presence of oxidative stress. Percentage change in radial growth was calculated by dividing a percentage of colony diameters of the deletion mutants over those of wild-type grown on the same plate. The data presented are the mean and standard deviation of two independent experiments with at least three replicates.

Chemical sensitivity assayed on PDA revealed that AaSSK1 deficient mutants were highly sensitive to H_2_O_2_, *tert*-butyl-hydroxyperoxide (*t*-BHP), and the superoxide (O_2_^-^)-producing agent menadione (MND) (Fig 2A), indicating the involvement of AaSSK1 in oxidative stress resistance. Fungal mutant impaired for a “two-component” histidine kinase (Δhsk1) [7] displayed wild-type resistance to test oxidants (Fig 2B). The mutant strain carrying defective HOG1 (Δhog1) [7] also was hypersensitive to H_2_O_2_, *t*-BHP and MND. The SKN7 deficient strain (Δskn7) displayed elevated sensitivity to H_2_O_2_, *t*-BHP, but not to MND, indicating that SKN7 was mainly involved in cellular resistance to peroxide, consistent with previous finding [8].

### AaSSK1 Is Required for Resistance to Osmotic Stress

**Fig 3:**
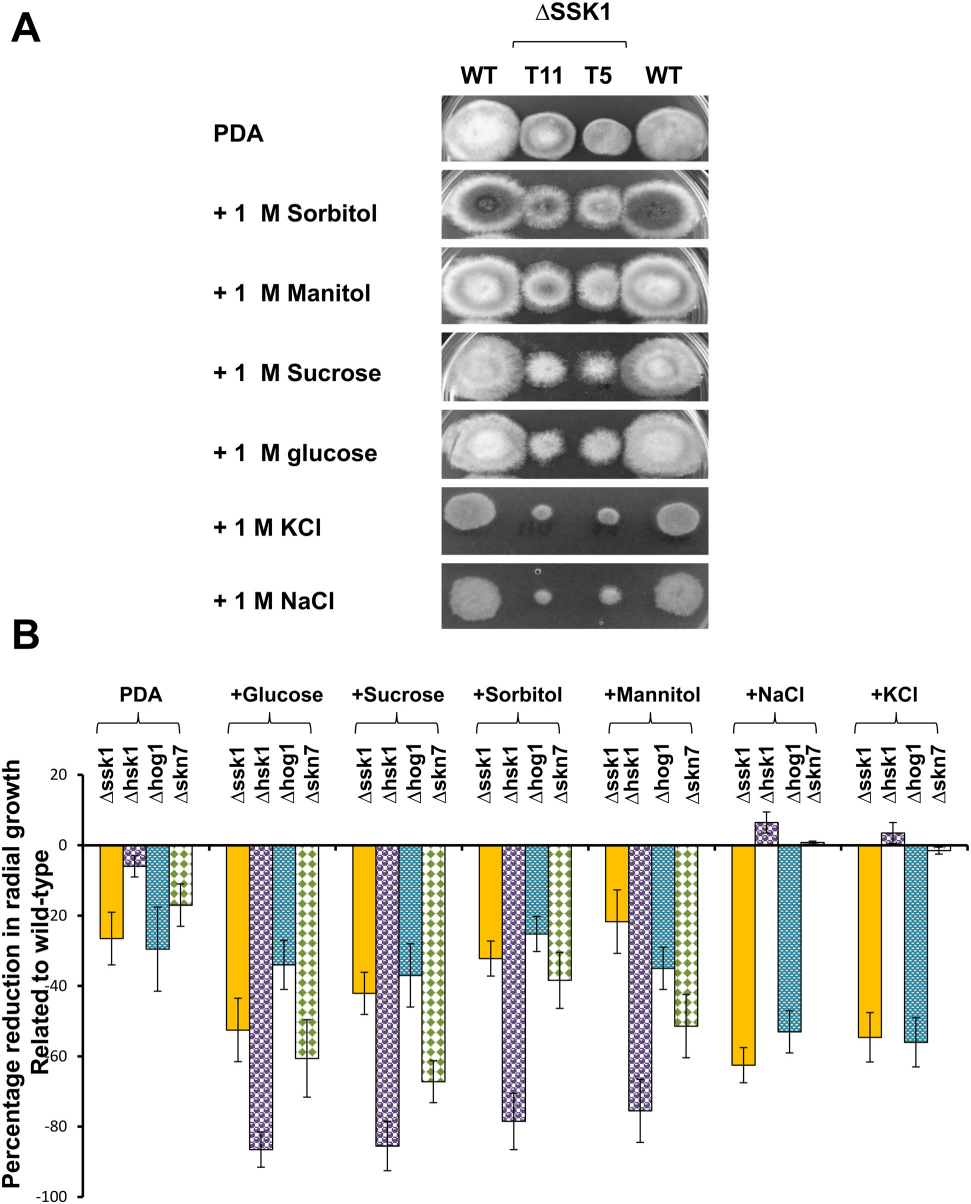
SSK1 plays a vital role in osmotic stress resistance in *A*. *alternata*. **(A)** Images of the wild-type (WT) and the Δssk1 deletion mutants (T5 and T11) grown potato dextrose agar (PDA) amended with sugars or salts for 4 to 5 days. **(B)** Quantitative comparison of Δssk1 with different mutant strains—Δhsk1 impaired for histidine kinase, Δhog1 for HOG1 MAP kinase and Δskn7 for SKN7 response regulator—in the presence of osmotic stress (each at 1 M). The data presented are the mean and standard deviation of two independent experiments with at least three replicates.

**Fig 4:**
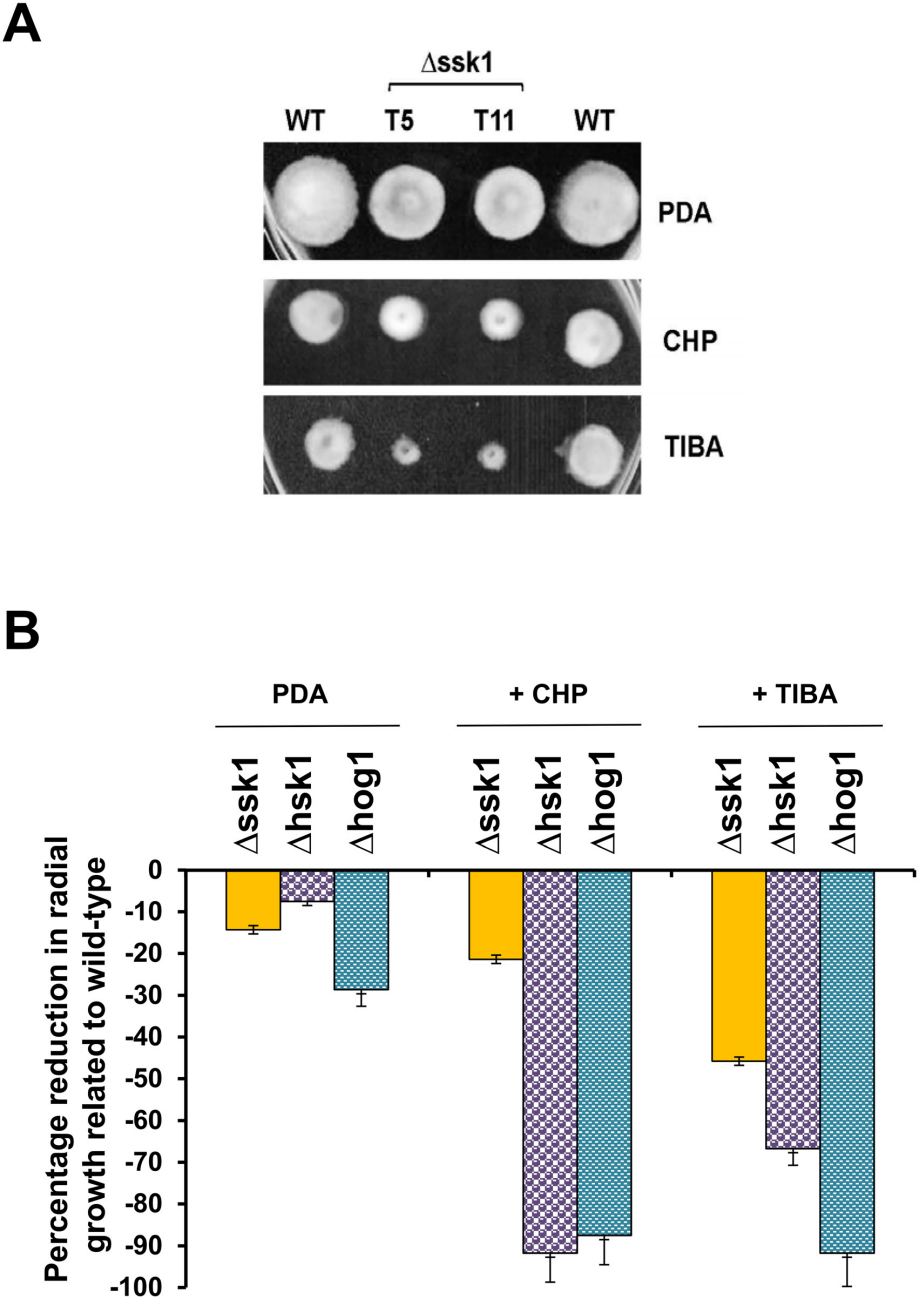
SSK1 is required for multi-drug resistance in *A*. *alternata*. **(A)** Images of the wild-type (WT) and the Δssk1 deletion mutants (T5 and T11) grown potato dextrose agar (PDA) amended with 2-chloro-5-hydroxypyridine (CHP) or 2,3,5-triiodobenzoic acid (TIBA), each at 5 mM. **(B)** Quantitative analysis of the wild-type, Δssk1, Δhog1, and Δhsk1 strains in the presence of CHP or TIBA.

Compared to wild-type, AaSSK1 deficient mutants displayed increased sensitivity to glucose, sucrose, KCl and NaCl (each at 1 M), indicating the involvement of AaSSK1 in osmotic stress resistance (Fig 3A). In contrast, Δhsk1 mutants displayed elevated sensitivity to glucose, sucrose, sorbitol, mannitol at levels greater than Δssk1 mutants and grew faster than wild type in the presence of KCl or NaCl (Fig 3B). Δhog1 mutants displayed wild-type sensitivity to sugars but displayed elevated sensitivity to KCl and NaCl at levels comparable to those of Δssk1 mutants. Previously, we found that deletion of a FUS3 MAP kinase-coding gene resulted in elevated resistance to 2,3,5-triiodobenzoic acid (TIBA) and 2-chloro-5-hydroxypyridine (CHP) [25]. TIBA is an auxin transport inhibitor and often used as a herbicide [26]. Pyridine containing a heteroaromatic ring could be found in many natural compounds [27]. Both Δhsk1 and Δhog1 were highly sensitive to TIBA and CHP, while Δssk1 displayed moderate sensitivity to these compounds (Fig 4). Δssk1 displayed wild-type sensitivity to cell wall biosynthesis inhibitors, calcofluor white and Congo red (data not shown).

### AaSSK1 Is Involved in H_2_O_2_ Detoxification

**Fig 5:**
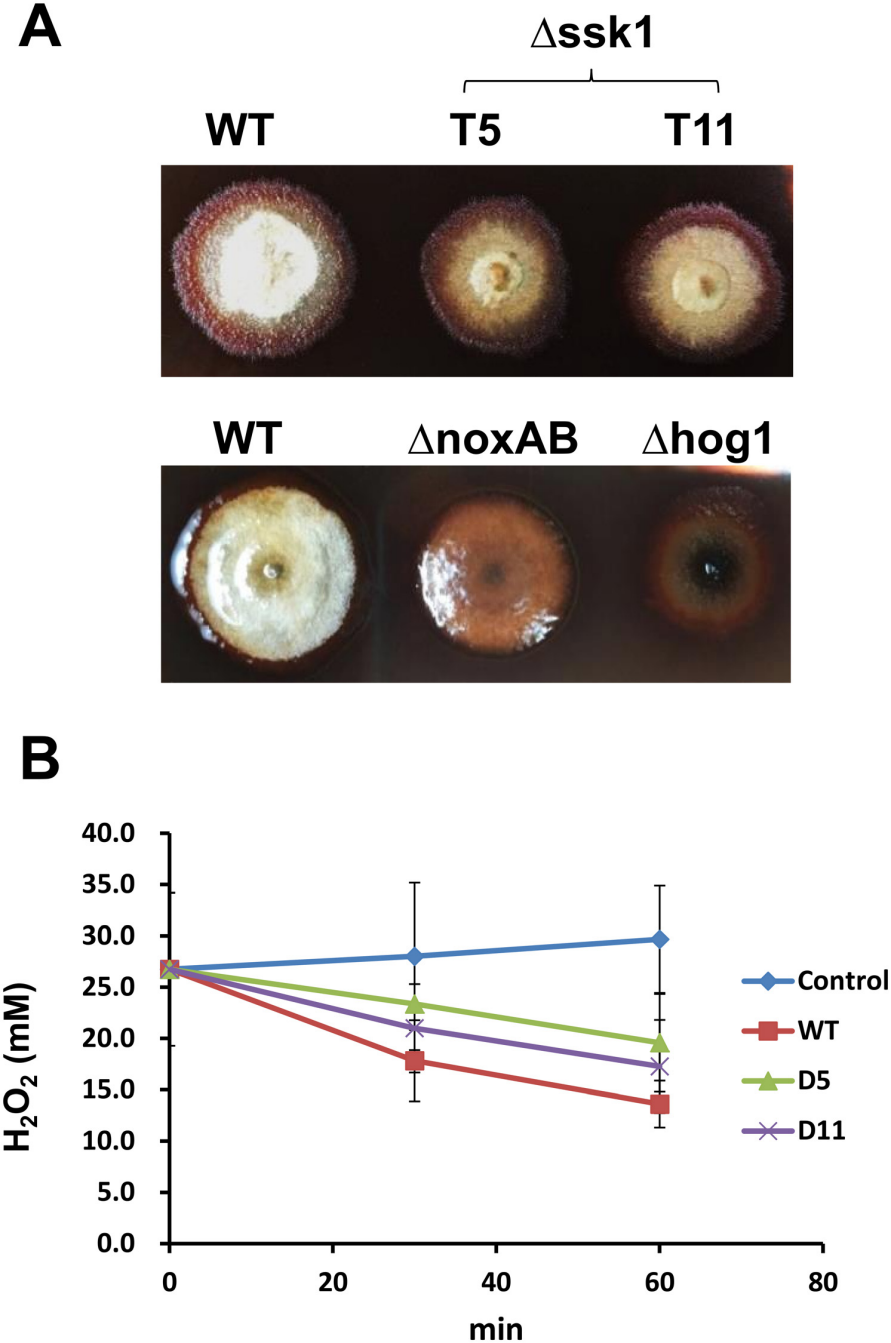
The *A*. *alternata* mutants impaired for *SSK1* are defective in the ability to detoxify H_2_O_2_. **(A)** The wild-type (WT) and the Δssk1 mutant strains (T5 and T11) grown on agar plates were flooded with 30 mM H_2_O_2_ and stained with 3,3’-diaminobenzidine. **(B)** Detoxification of H_2_O_2_ by *A*. *alternata* strains was determined by monitoring a decrease of absorbance at 240 nm over time. Five agar plugs (2 mm in diam.) containing fungal conidia/hyphae were cut from fungal colonies grown on PDA for 3 to 5 days, placed in solution, and incubated at ~25°C. The control treatment contained no fungal hyphae. The data presented are the mean and standard deviation of three independent experiments with three replicates.

The ability of *A*. *alternata* strains to detoxify H_2_O_2_ was assessed by growing them on PDA plates for 4 days. Fungal colonies were submerged in 30 mM H_2_O_2_ and stained with DAB, which reacted with H_2_O_2_, accumulating a brownish polymer around fungal colonies. Fungal colonies became brown 6 h after staining with DAB, indicative of the accumulation of H_2_O_2_ around mutant strains. Δssk1 mutant colonies proliferate slowly after 24 h incubation failing to produce fluffy mycelium (Fig 5A). In contrast, only the edge of wild-type colony was surrounded with brownish pigments after treating with H_2_O_2_ and staining with DAB. Wild-type colony continued to produce white mycelium after 24 h incubation. Colonies of the mutant strains carrying defective HOG1 (Δhog1) or impaired for NADPH oxidases NOXA and NOXB (ΔnoxAB double mutant) [11] became dark-brown after treating with H_2_O_2_ and staining with DAB and failed to recover and produce fluffy mycelium after prolonged incubation. Degradation of H_2_O_2_ by *Alternaria* strains was further determined spectrophotometrically by measuring the absorbance at 240 nm for the amount of H_2_O_2_ decreasing over time in solution without adding UV chromophores [28]. The results revealed that AaSSK1 deficient mutants degraded H_2_O_2_ at rates much slower than that of wild-type, indicating an impairment of ROS detoxification ability in Δssk1 (Fig 5B).

### AaSSK1 Impaired Mutants Exhibit Increased Resistance to Fungicides

**Fig 6:**
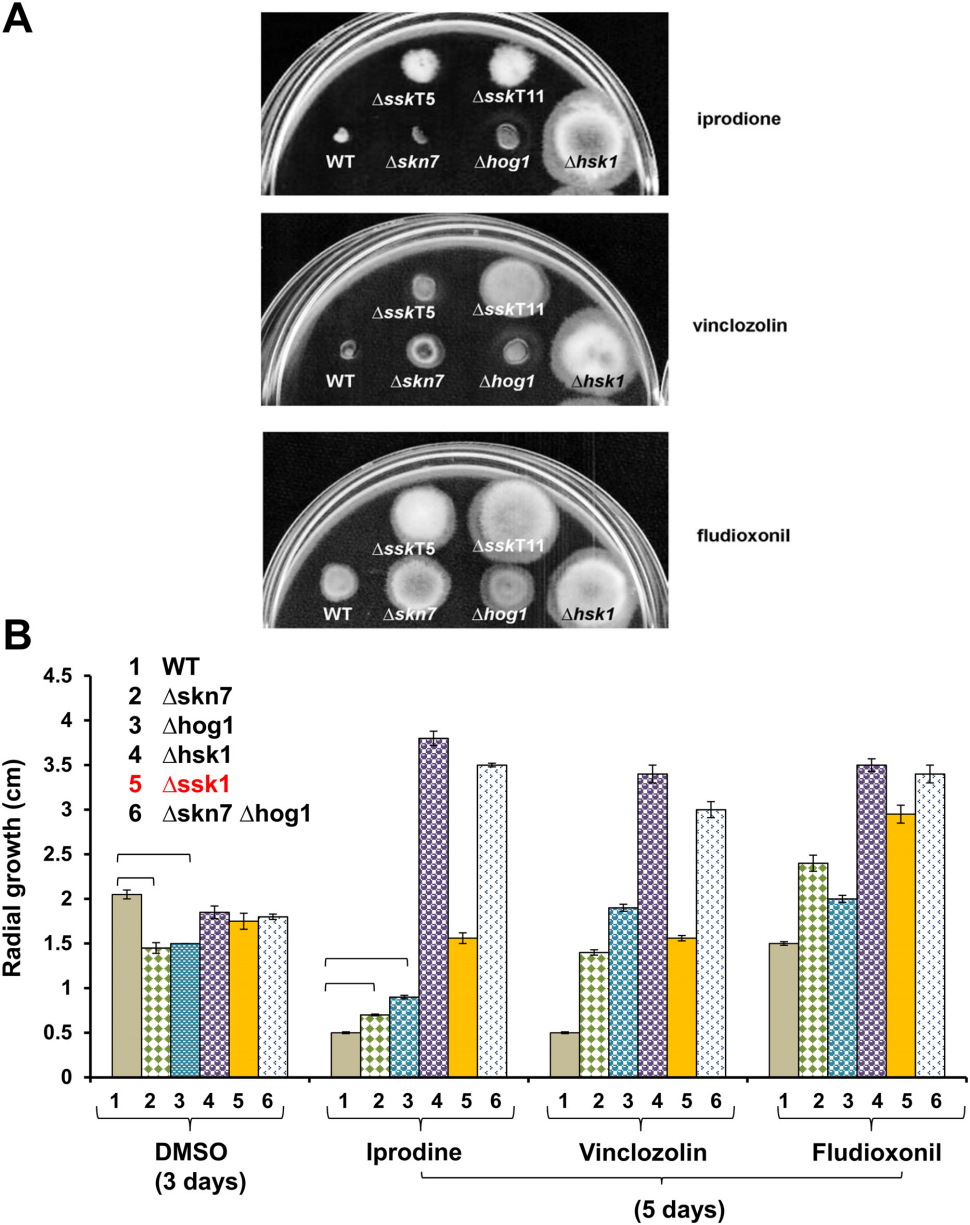
The *A*. *alternata* mutants confer resistance to fungicides. **(A)** Growth of the wild-type (WT), the strains lacking *SSK1* (*Δ*ssk1-T5 and *Δ*ssk1-T11), the histidine kinase (*Δ*hsk1), the HOG1 MAP kinase (*Δ*hog1), and the *SKN7* response regulator (*Δ*skn7) on PDA amended with vinclozolin (10 μg/ml), fludioxonil (0.1 μg/ml) or iprodione (10 μg/ml) dissolved in 1% DMSO for 3 to 5 days. **(B)** Quantitative analysis of fungal radial growth. The *Δ*skn7 *Δ*hog1 strain was impaired for both *SKN7* and *HOG1*. Percentage change in radial growth was calculated by dividing a percentage of colony diameters of the deletion mutants over those of wild-type grown on the same plate. The data presented are the mean and standard deviation of two independent experiments with at least three replicates. Top Brackets indicate significant differences between treatments.

*A*. *alternata* was highly sensitive to dicarboximide (iprodione and vinclozolin) and phenylpyrrole (fludioxonil) fungicides (Fig 6). Δhsk1 was highly resistant to these fungicides. Disruption of SSK1, HOG1 or SKN7 also resulted in fungal strains that displayed increased resistance to these fungicides to varying levels. Fungal strain impaired for either HOG1 or SKN7 displayed increased resistance to fungicides at levels lower than Δhsk1. However, mutant strain impaired for both HOG1 and SKN7 displayed fungicide resistance comparable to Δhsk1. Δssk1 mutants displayed fungicide resistance at levels very much less than Δhsk1 and Δhog1 Δskn7 double mutants, but greater than mutants mutated at HOG1 or SKN7 alone.

### AaSSK1 Is Required for Fungal Virulence

**Fig 7:**
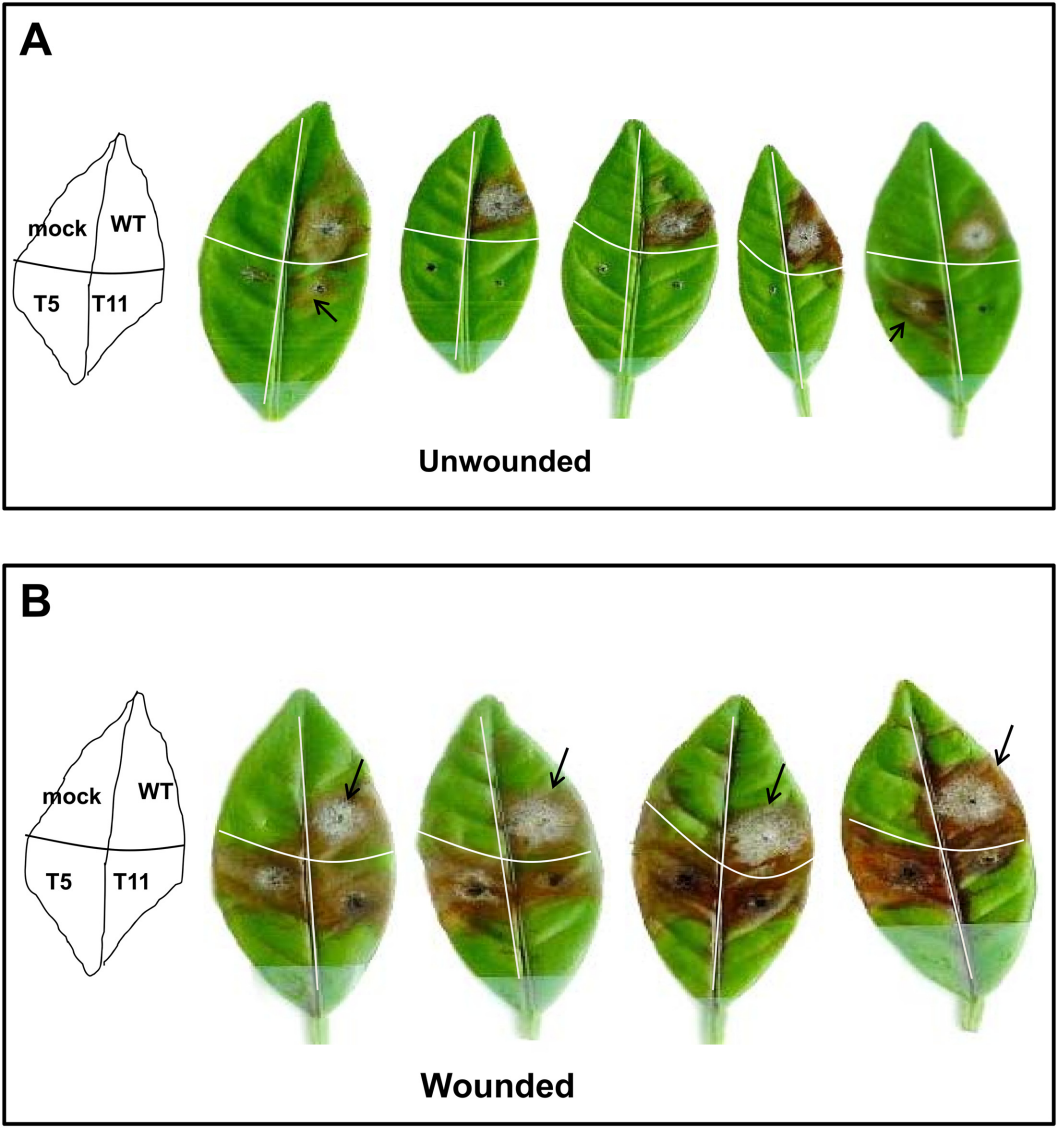
SSK1 plays a crucial role in *A*. *alternata* pathogenesis in citrus. **(A)** Development of necrotic lesions on detached calamondin leaves inoculated with conidial suspension (10^4^ conidia per ml) prepared form the wild-type (WT) and the *Δ*ssk1 mutant strains (T5 and T11). The mock controls were treated with water only. *Δ*ssk1 mutants occasionally induced visible lesions (indicated by arrows). **(B)** Induction of necrotic lesions by *A*. *alternata* strains on detached calamondin leaves that were wounded prior to inoculation. Inoculated leaves were kept in plastic boxes for 5 days. Wild-type produced aerial hyphae and conidia on necrotic areas (indicated by arrows).

Fungal infection assays on detached calamondin leaves revealed a reduction of necrotic lesions induced by the *A*. *alternata SSK1* mutant, as compared to those induced by wild-type. Late in pathogenesis wild-type caused disease symptoms that included the production of large spreading lesions across the leaf surface and necrosis (Fig 7). All calamondin leaves (N = 15) inoculated with conidial suspensions of wild-type produced necrotic lesions where it proliferated, formed colonies, and produced visible aerial hyphae 5 days post inoculation (dpi). Leaves inoculated with conidial suspensions prepared from Δssk1 rarely resulted in visible lesions. If lesions were ever produced by Δssk1, the mutants seemingly proliferated slower, produced much smaller colonies, and formed fewer aerial hyphae than wild-type on necrotic areas. When citrus leaves were wounded prior to inoculation, Δssk1 mutants induced necrotic lesions in all inoculated spots; however, fungal proliferation was reduced as the mutants produced smaller colonies on necrotic lesions than wild-type.

### Disruption of *AaSHO1* Results in No Noticeable Phenotypes

Two *A*. *alternata* mutants (Δsho1-T46 and Δsho1-T59) impaired for *SHO1* were identified (S2 Fig). The mutants displayed wild-type sensitivity to H_2_O_2_, oxidants, sugars, and salts, indicating that AaSHO1 played no roles in oxidative and osmotic stress resistance (S3 Fig). Unlike mutant strains impaired for SSK1, Δsho1 mutants displayed wild-type sensitivity to iprodione, vinclozolin and fludioxonil fungicides, calcofluor white and Congo red, TIBA and CHP. Moreover, unwounded citrus leaves (N = 20) inoculated with Δsho1 mutants developed necrotic lesions similar to those induced by wild-type (data not shown), indicating no roles of SHO1 in *A*. *alternata* pathogenesis.

## Discussion

The hypersensitive reaction (HR), characterized by the accumulation of toxic reactive oxygen species (ROS), plays a critical role in plant defense machinery against saprophytic microorganisms and biotrophic phytopathogens, yet has less effect against necrotrophic plant pathogens [29]. Necrotrophic fungi often deploy toxins or cell wall-degrading enzymes to kill host cells prior to colonization and thus are less affected by the HR. Indeed, necrotrophic fungi may utilize ROS-induced damages to their advantage [30]. The tangerine pathotype of *A*. *alternata* produces the host-selective ACT toxin that kills host cells before establishment [2]. *A*. *alternata* infection in citrus leaves induces rapid lipid peroxidation, accumulation of hydrogen peroxide (H_2_O_2_) and cell death [31].

In this paper we report that the *A*. *alternata SSK1* (*AaSSK1*) is a key positive regulator of environmental stimulants, particularly with regard to oxidative and osmotic stress. AaSSK1 acts as a regulator of resistance to toxic ROS, has tolerance to high concentrations of salt and to a lesser degree sugars, and allows for full virulence of the fugal pathogen in citrus host. Experimental results show that AaSSK1 is one of the cellular targets of iprodione, vinclozolin and fludioxonil fungicides. We also demonstrate the requirement of AaSSK1 in fungal development. This diversity of AaSSK1 function implicates the complexity of environmental cues by which fungal ability to cope with ominous stress is subtly regulated. Although AaSSK1 plays no roles in the quantity of conidia, deletion of *AaSSK1* in *A*. *alternata* causes severe reduction in conidial germination as assayed on 96-well microtiter plates. Deletion of *AaSSK1* also affects the pattern of conidial germination. Δssk1 mutants predominately produced single germ tubes and only a few Δssk1 conidia germinated and produced more than two germ tubes. On the contrary, the majority of wild-type conidia germinated and produced multiple germ tubes, suggesting a critical requirement of *AaSSK1* in fungal differentiation. Deletion of a phospholipase C-encoding gene (*PLC1*) in *A*. *alternata* also affected hyphal elongation, conidial formation and germination pattern of conidia [32], suggesting a connection between the AaSSK1-mediated signaling pathways and the PLC1-mediated Ca^2+^ homeostasis in terms of conidial development.

In this work, we have also shown that the predicted *AaSSK1* gene product has characteristic features of a stress response regulator. These include conservation of a signal receiver domain and amino acids involved in phosphorylation, recognition, and dimerization, and conservation of a stress responsive element (STRE: AGAGGGG) found in the 5’ untranslated region of *AaSSK1*. AaSSK1 also show a high degree of amino acid similarity, including a large set of identical amino acids, to numerous response regulators found in the yeasts and filamentous fungi. Δssk1 null mutants have a ROS-sensitive phenotype, similar to that observed in *rrg1* mutants of *Fusarium graminearum* and *Botrytis cinerea* [19,20]. However, *Aspergillus nidulans* and *Magnaporthe oryzae* mutants lacking *SSK1* homologs display wild-type sensitivity to H_2_O_2_ [33,34]. The finding that Δssk1 mutants are impaired in their ability to cope with environmental stress is consistent with the prediction that AaSSK1 is involved in stress response and regulation.

**Fig 8:**
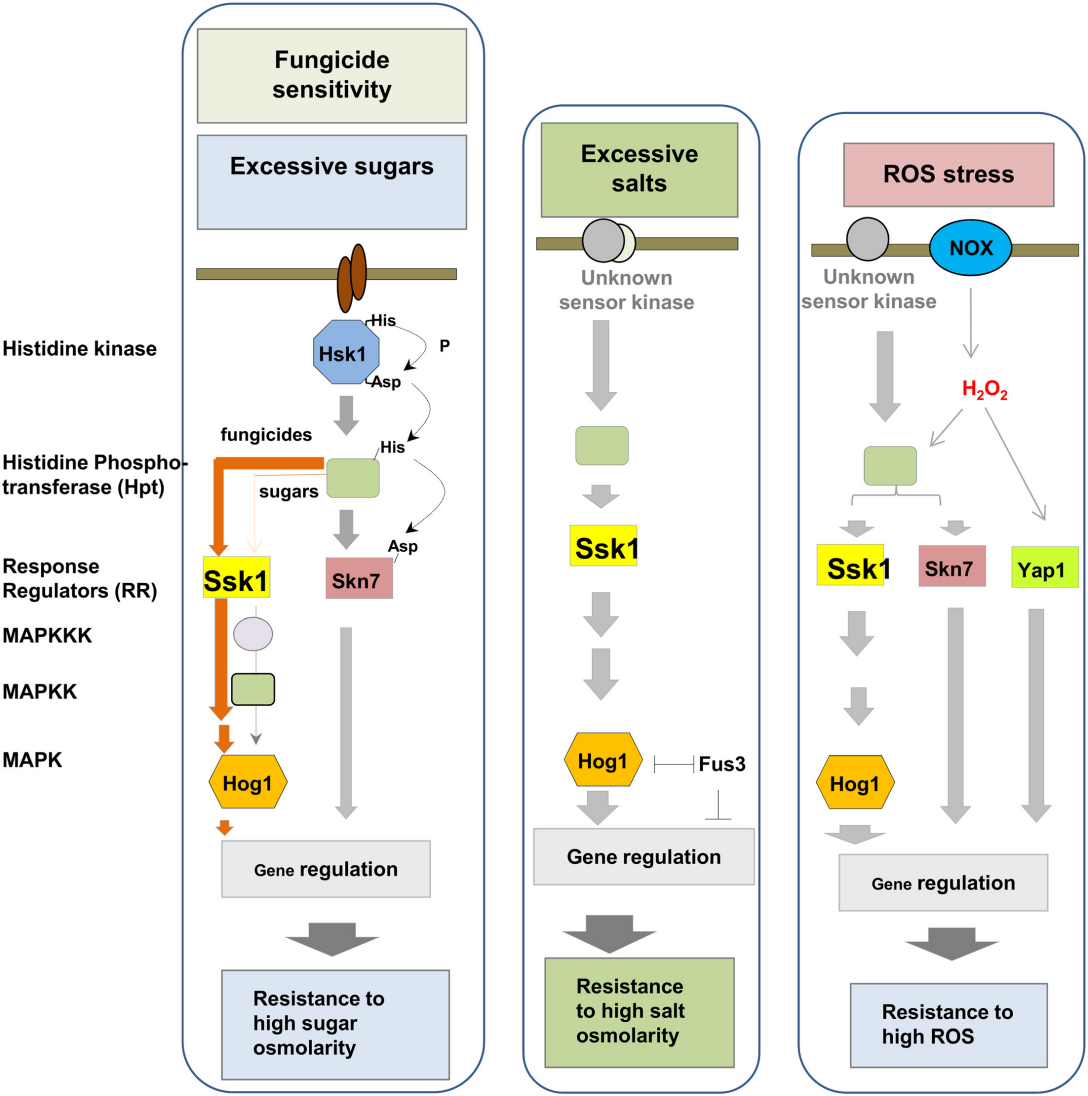
Schematic illustration of signaling pathways leading to osmotic and oxidative stress resistance and fungicide sensitivity in *A*. *alternata*. *A*. *alternata* deploys the HSK1- and the SKN7-mediated phosphorelay signaling pathways between histidine (His) and asparate (Asp) to cope with sugar-induced osmotic stress. SSK1 plays a minor role in sugar-induced osmotic stress resistance. However, SSK1 and HOG1 mediated by unknown kinase sensors confer resistance to oxidative and salt-induced osmotic stress. Low-level H_2_O_2_ produced by NADPH oxidases (NOX) is proposed to activate SSK1, HOG1, YAP1, and SKN7, all implicated in ROS resistance. HSK1, SSK1, HOG1, SKN7, but not YAP1 and NOX are involved in fungicide sensitivity.

*A*. *alternata* is capable of dealing with toxic ROS via multiple regulatory pathways. The ability to alleviate oxidative stress involving the YAP1 transcription regulator, the HOG1 MAP kinase, the SKN7 regulator and the NADPH oxidase (NOX) plays a crucial role in the pathogenesis of *A*. *alternata* in citrus [5,7−9,11]. SSK1 may independently and cooperatively interact with those regulators in the context of ROS resistance and osmoadaptation. The NOX complex has a dual function in ROS production and resistance. It has been thought that the NOX-produced H_2_O_2_ acts as a signal to activate YAP1, SKN7, HOG1 and perhaps SSK1 regulators, which result in the further activation of downstream genes in response to oxidative stress (Fig 8). Expression of both *YAP1* and *HOG1* genes are regulated by NOX-produced H_2_O_2_, while overexpression of *YAP1* and *HOG1* suppresses the expression of the NOX-coding genes [11], implicating a transcriptional feedback inhibition. Although both SSK1 and SKN7 are involved in H_2_O_2_ resistance, the *A*. *alternata* SKN7 plays no roles in superoxide resistance. *A*. *alternata* mutant lacking SKN7 displays wild-type resistance to superoxide-generating compounds such as potassium superoxide, menadione and diamide [8]. In contrast, SSK1 and its downstream HOG1 MAP kinase [7] are responsible for resistance to both H_2_O_2_ and superoxide. The inability to detoxify toxic ROS seen in the Δssk1 mutants is probably due to decreased activities of antioxidant enzymes. Deletion of the *YAP1* or *SKN7* gene results in a reduction of peroxidase, catalase and superoxide dismutase (SOD) activities, all implicated in ROS detoxification [8,31]. Moreover, activation of SSK1 and SKN7 in response to ROS is unlikely mediated by “two component” histidine kinase (HSK1) because the HSK1 mutant displays wild-type resistance to ROS. Thus *A*. *alternata* could also deploy unknown sensors other than HSK1 to sense environmental oxidative stress (Fig 8).

Apart from ROS resistance, an important role for a SSK1 response regulator in cellular tolerance to osmotic stress also was demonstrated in the present study. *A*. *alternata* mutants impaired for SSK1 display increased hypersensitivity to NaCl or KCl, a phenotype highly resembling the mutants lacking HOG1 [7]. Compared to wild-type, Δssk1 mutants also display moderate sensitivity to glucose and sucrose, and display no sensitivity to sorbitol and mannitol. *A*. *alternata* mutants lacking HOG1 retain wild-type levels of resistance to sugars. The involvement of SSK1 in osmotic stress resistance is supported further by the fact that the Δssk1 mutants produced very few or no protoplasts after incubating with cell wall-degrading enzymes in a NaCl-containing osmotic solution [35]. Deletion of *HOG1* in *A*. *alternata* also failed to generate viable protoplasts [36]. Because mutants impaired for HSK1 are highly sensitive to sugars but not salts, it appears that *A*. *alternata* dealing with salt-induced stress via SSK1 and HOG1 is likely via an unknown kinase sensor rather than HSK1 (Fig 8). Moreover, the FUS3 MAP kinase-mediated signaling pathway negatively regulates cellular adaptation to salt-induced stress likely via counteracting HOG1 in *A*. *alternata*, as FUS3 mutant displays elevated resistance to both NaCl and KCl and increases the accumulation of both *HOG1* gene transcript and HOG1 protein phosphorylation, and vice versa [25,36]. While the presence of the *SSK1* gene contributes to osmotic stress resistance, it may not be the only one. To deal with sugar-induced osmotic stress, *A*. *alternata* mainly utilizes the HSK1- and the SKN7-mediated signaling pathways (Fig 8), as deletion of *HSK1* or *SKN7* results in fungi displaying increased sensitivity to sugars. In contrast, SSK1 and HOG1 play moderate roles in sugar-induced stress. As shown in Fig 4, fungal strains lacking *HSK1*, *SSK1* or *HOG1* are hypersensitive to TIBA and CHP, suggesting a role of the HSK1-SSK1-HOG1 signaling pathway in multi-drug resistance. Overall, our results have demonstrated that disruption of *AaSSK1* impacts vegetative growth, conidial germination, differentiation and protoplast formation in *A*. *alternata*.

*A*. *alternata* is highly sensitive to vinclozolin, iprodione and fludioxonil fungicides. However, *A*. *alternata* mutants defective in SSK1, HSK1, HOG1 or SKN7 are all resistant to those fungicides [7,8], suggesting possible interactions among these signaling components in terms of fungicide sensitivity. It becomes apparent that unlike cellular response to osmotic stress, the involvement of both SSK1 and SKN7 in fungicide sensitivity is likely mediated by the HSK1 signaling pathway in *A*. *alternata* (Fig 8). Intriguingly, vinclozolin, iprodione and fludioxonil fungicides also affect a glutathione peroxidase GPx3 and a NOX component NOXB. Fungal strains mutated at GPx3 or NOXB, but not NOXA or NOXR, are resistant to these fungicides [11,12]. *AaSSK1* mutant strains display elevated resistance to vinclozolin, iprodione and fludioxonil fungicides, suggesting that SSK1 is one of the targets of these fungicides. Both dicarboximide and phenylpyrrole fungicides have been shown to impact the Group III histidine kinase and HOG1-mediated signaling pathways in fungi [37].

Although a generalized role for a SSK1-like response regulator in signaling transduction pathway in yeasts has been well studied [16,38], this regulation would not readily define the actual role of SSK1 in *A*. *alternata* pathogenesis. In *S*. *cerevisiae*, SSK1 is one of the primary osmotic regulators. However, the *A*. *alternata* SSK1 is involved in ROS resistance, osmotic resistance, fungicide sensitivity and fungal virulence. AaSSK1 plays an important role in the establishment of plant colonization because gene deletion mutants of *SSK1* are reduced in their ability to induce necrotic lesions on susceptible citrus cultivar. Although the slow growth rate of Δssk1 mutants may lead to decreased colonization of host tissue, Δssk1 mutants also display elevated sensitivity to ROS and rarely induce necrotic lesions on susceptible citrus leaves. When citrus leaves were wounded prior to inoculation, the Δssk1 mutants induced necrotic lesions at rates slower than those induced by wild-type, formed smaller colonies and produced fewer aerial hyphae than wild-type on necrotic areas, indicating a vital role of SSK1 in fungal penetration and colonization. The results are consistent with previous finding that the ability of ROS detoxification and resistance is absolutely required for *A*. *alternata* pathogenesis in citrus [5−8]. An analysis of the SSK1-regulated genes may lead to a better understanding how AaSSK1 is capable of having such diverse functions in developmental processes, stress resistance and pathogenesis.

In the budding yeast *S*. *cerevisiae*, high osmolarity activates both SLN1 (a “two component” histidine kinase) and SHO1 that independently regulate HOG1 [17]. Activated HOG1 in turn phosphorylates SHO1 [39]. SHO1 homologs have been shown to play important roles in developmental and pathological processes in various plant pathogenic fungi, including *Fusarium oxysporum* and *F*. *graminearum* [40–43]. In contrast, disruption of *AaSHO1* results in no noticeable phenotypes, suggesting that AaSHO1 plays a little or no role in osmotic and oxidative stress resistance in the tangerine pathotype of *A*. *alternata*. Alternatively, SHO1 and SSK1 may have redundant functions in which SSK1 compensates for stress deficiencies seen in the *SHO1* deletion mutant. In yeasts, SHO1 and SLN1 (an upstream regulator of SSK1) are functionally redundant; either SHO1 or SLN1 is sufficient to survive in high osmolarity conditions [44]. Attempts to create double mutants defective for both *SSK1* and *SHO1* in *A*. *alternata* failed, suggesting that deletion of both genes could be lethal. The actual function of *SHO1* in the tangerine pathotype of *A*. *alternata* remains elusive.

## Materials and Methods

### Fungal Strains and Culture Conditions

The wild-type EV-MIL31 strain of *Alternaria alternata* (Fr.) Keissler was isolated from diseased citrus leaves [5] and used for transformation and mutagenesis. Fungal strains defective for a SKN7 response regulator (Δskn7), a high osmolarity glycerol (Δhog1) mitogen-activated protein kinase, and a “two component” histidine kinase (Δhsk1) were created from the EV-MIL31 strain in previous studies [7,8]. Unless otherwise stated all fungal strains were grown on potato dextrose agar (PDA) (Difco, Sparks, MD) at 28°C. Fungal mycelium was ground in sterile water using a disposable pestle (Fisher Scientific, Atlanta, GA), evenly spread onto a layer of cellophane overlaid on PDA for 3 to 5 days, and harvested for DNA or RNA isolation. For conidia formation, fungal strains were grown on PDA without parafilm sealing in light for 5 days, and harvested by immersing, scraping with sterile water, passing through Miracloth and centrifugation (5,000 xg). Conidia were examined with a Zeiss Axio Imager A1 microscope equipped with an AxioCam MRm CCD camera (Oberkochen, Germany). Germination of conidia was assessed by placing them on glass slides or 96-well microtiter plates and incubating in a plastic box for 6 h. Fungal hyphae were treated with cell wall degrading enzymes to release protoplasts in a solution containing sucrose as an osmotic agent as described [35].

### Assays for Chemical Sensitivity

Assays for chemical sensitivity were performed by transferring conidia/ hyphae using a sterile toothpick onto PDA containing the test compound. The diameter of colony was recorded at 4 to 7 days after incubation at room temperature (~25°C). Changes of radial growth (%) were determined as a cumulative percentage of growth of wild-type and mutant strains cultured on the same plate. Vinclozolin (10 μg/ml), iprodione (10 μg/ml) and fludioxonil (0.1 μg/ml) fungicides (Sigma, St. Louis, MO) were dissolved in 1% DMSO; other chemicals were dissolved in water, filter sterilized, and added to medium. All experiments were repeated at least two times with duplicate of each treatment.

### Targeted Gene Deletion

The *A*. *alternata SSK1* (*AaSSK1*) and *SHO1* (*AaSHO1*) genes were deleted by a split marker approach as described [45]. A bacterial phosphotransferase B gene (*HYG*) cassette under control of the *Aspergillus nidulans trpC* gene promoter and terminator conferring resistance to hygromycin was split by PCR amplification and fused with truncated *SSK1* (or *SHO1*) fragments as shown in S1 and S2 Figs. PCR products (10 μl each) were combined and transformed into fungal protoplasts prepared from the EV-MIL31 strain using CaCl_2_ and polyethylene glycol as previously described [35]. Fungal transformants appearing on a regeneration medium amended with 200 μg/ml hygromycin (Roche Applied Science) were picked and screened by PCR with different pairs of *SSK1* or *SHO1* gene-specific primers as indicated in S1 and S2 Figs. The SSK1 R2(-) primer that is not present in the split marker fragments was paired with the hyg4 primer and used to examine for the occurrence of homologous integration within *SSK1*. A 2.2-kb fragment was amplified with the primers SSK1 R2(-) and hyg4 from genomic DNA prepared from transformants T5, T9 and T11, while no product was amplified from that of wild-type (S1 Fig). A 1.0 kb fragment was amplified with the primers SSK10 (+) and SSK1 R2 (-) from genomic DNA of wild-type while no product was obtained from those of T5, T9 and T11, indicating that they were impaired at *SSK1*. The primer Sho1 F2 (+), which is not present in the split marker fragments was paired with hyg3 and amplified a 2.7-kb fragment from transformants T46, T53 and T59 and no product was amplified from that of wild type (S2 Fig). The primers Sho1 F1 (+) and Sho1 R1(-) amplified a 3.1-kb fragment from wild-type and a 5-kb fragment from transformants T46 and T59, indicating that both T46 and T59 were impaired at *SHO1*. Fungal DNA was isolated using a DNeasy Plant kit (Qiagen, Valencia, CA). PCR fragments were purified using a PCR Purification kit (GenScript, Piscataway, NJ) and sequenced at Eton Bioscience (Research Triangle Park, NC). The exon/intron positions were determined based on the deduced protein sequence, which was obtained from similarity search using the Blastx program available at the National Center for Biotechnology Information (NCBI) website. Oligonucleotide primers used in this study are reported in S1 Table.

### Assays for Fungal Virulence

Fungal virulence was assessed on detached calamondin (*Citrus mitis* Blanco) leaves inoculated by placing 10 μl of conidial suspension (10^4^ conidia per ml) on each spot as described previously [5,9]. The inoculated leaves were incubated in a plastic box for 5 days for lesion development. Each fungal strain was tested on at least 15 leaves and experiments were repeated three times.

### Detoxification of H_2_O_2_

Detoxification of H_2_O_2_ by *Alternaria alternata* was carried out in 2-ml water containing 30 mM H_2_O_2_ with or without fungal strain as described previously [8]. Five agar plugs (2 mm in diam.) containing fungal conidia/hyphae were cut from fungal colonies grown on PDA for 3 to 5 days, placed in solution, and incubated at room temperature (~25°C). H_2_O_2_ was monitored spectrophotometrically at A_240_ nm over time [28]. The control treatment contains no fungal hyphae. Detoxification of H_2_O_2_ by *A*. *alternata* strains also was assessed on agar plates. Fungal strains were cultured on PDA for 4 days, immersed in 30 mM H_2_O_2_ for 30 min, drained, flooded with 5% 3,3’-diaminobenzidine (DAB), and incubated for an additional 24 h. DAB preferably reacts with H_2_O_2_ to form a brownish polymer. The formation of brown color, indicative of the presence of H_2_O_2_ was recorded.

### Nucleotide Sequence

Sequence data reported in this article have been deposited in the GenBank/ EMBL Data Libraries under Accession Nos. KU170060 (*AaSSK1*) and KU170061 (*AaSHO1*).

### Ethics Statement

No ethical permissions were required for this work which involved no experimentation involving animals or human samples.

## Supporting Information

S1 Fig Targeted disruption of *AaSSK1* using a split marker approach. (DOCX)

S2 Fig Targeted disruption of *AaSHO1* using a split marker approach. (DOCX)

S3 Fig Chemical sensitivity tests. (DOCX)

S1 Table Oligonucleotide primers used in this study. (DOCX)
