# Supplementary material for: How the Pathogenic Fungus Alternaria alternata Copes with Stress via the Response Regulators SSK1 and SHO1
Source: PLoS One. 2016 Feb 10;11(2):e0149153. doi: 10.1371/journal.pone.0149153 (PMC4749125; doi:10.1371/journal.pone.0149153)
Supplement: S2 Fig — (DOCX) [file pone.0149153.s002.docx]

**Supporting Information**

**S2 Fig. Targeted disruption of *AaSHO1* using a split marker approach.**

**A.** Schematic depiction of generation of truncated but overlapping hygromycin phosphotransferase gene (*HYG*) under control by the *Aspergillus nidulans* *trpC* promoter (P) and terminator (T) within *AaSHO1*. Oligonucleotide primers used to amplify each fragment are indicated.

**B.** Image of DNA fragments amplified from genomic DNA of wild type (WT) and transformants with the primer SHO1F2(+) paired with hyg3.

**
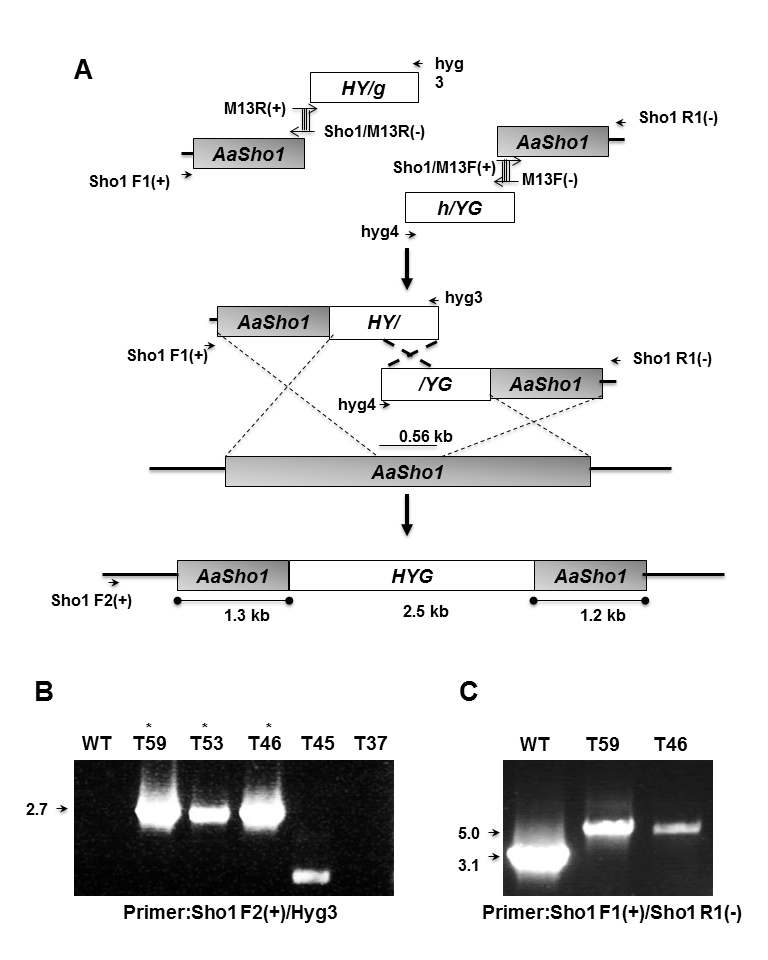
C.** Image of DNA fragments amplified from genomic DNA of wild type (WT) and transformants with the primer SHO1F1(+) paired with SHO1R1(-) indicating that *AaSHO1* is deleted in transformants T46 and T59.
