# Supplementary material for: How the Pathogenic Fungus Alternaria alternata Copes with Stress via the Response Regulators SSK1 and SHO1
Source: PLoS One. 2016 Feb 10;11(2):e0149153. doi: 10.1371/journal.pone.0149153 (PMC4749125; doi:10.1371/journal.pone.0149153)
Supplement: S3 Fig — (DOCX) [file pone.0149153.s003.docx]

**Supporting Information**

**S3 Fig. Chemical sensitivity tests.**

Images of the wild-type (WT) and the ∆sho1 deletion mutants (T46 and T59) grown potato dextrose agar (PDA) amended with different chemicals as indicated for 4 to 5 days. Abbreviation: CHP, 2-chloro-5-hydroxypyridine and TIBA, 2,3,5-triiodobenzoic acid.

**
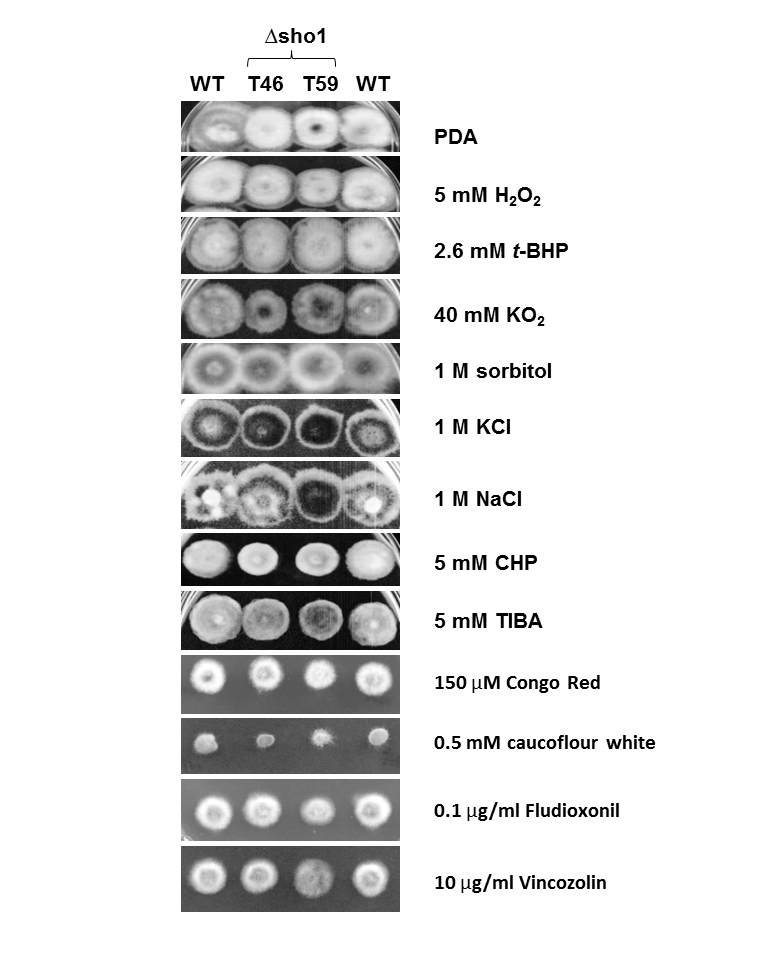
**
