# Supplementary material for: How the Pathogenic Fungus Alternaria alternata Copes with Stress via the Response Regulators SSK1 and SHO1
Source: PLoS One. 2016 Feb 10;11(2):e0149153. doi: 10.1371/journal.pone.0149153 (PMC4749125; doi:10.1371/journal.pone.0149153)
Supplement: S1 Table — (DOCX) [file pone.0149153.s004.docx]

**Supporting Information**

**S1 Table. Oligonucleotide primers used in this study.**

| Primer | Sequence (5’-3’) | Corresponding gene and remarks |
| --- | --- | --- |
| M13R | agcggataacaatttcacacagga | pUCATPH1 |
| M13F | cgccagggttttcccagtcacgac | pUCATPH1 |
| hyg3 | ggatgcctccgctcgaagta | *Hyg^r^* |
| hyg4 | cgttgcaagacctgcctgaa | *Hyg^r^* |
| SSK10 (+) | atcaaggcgcggctgcacaa | *SSK1* forward |
| SSK1 R2 (-) | gatgataagggcctcgtgga | *SSK1* reverse |
| SSK1/M13R(-) | tcctgtgtgaaattgttatccgctgacccagatcttgcggtgca | *SSK1* carrying sequence (underlined) complementary to M13R primer |
| SSK1/M13F(+) | gtcgtgactgggaaaaccctggcgactcccttcgcccggcggcac | *SSK1* carrying sequence (underlined) complementary to M13F primer |
| SRR R1(-) | caccgaaccttcaaacgctg | *SSK1* reverse |
| SRR R2(-) | gagcttcgatgtggtgt | *SSK1* reverse |
| Sho1 F1(+) | tagtcgttgaaagtgaccca | *SHO1* forward |
| Sho1/M13R(-) | tcctgtgtgaaattgttatccgctccattttgcgtagtgaagga | *SHO1* carrying sequence (underlined) complementary to M13R primer |
| Sho1/M13F(+) | gtcgtgactgggaaaaccctggcgcgacagattgtaggcttcct | *SHO1* carrying sequence (underlined) complementary to M13F primer |
| Sho1 R1(-) | ctccatgtgtgtctctctct | *SHO1* reverse |
| Sho1 F2(+) | cagaagtggagtgaagagtc | *SHO1* forward |

pUCATPH1, a plasmid harboring a hygromycin resistance cassette

Hyg^r^, a bacterial phosphotransferase B gene (*HYG*) cassette conferring hygromycin resistance
